# Standard Lexicons, Coding Systems and Ontologies for Interoperability and Semantic Computation in Imaging

**DOI:** 10.1007/s10278-018-0069-8

**Published:** 2018-05-03

**Authors:** Kenneth C. Wang

**Affiliations:** 1 Baltimore VA Medical Center, 10 N. Greene St., Room C1-24, Baltimore, MD 21201 USA; 2 Department of Diagnostic Radiology and Nuclear Medicine, University of Maryland School of Medicine, Baltimore, USA

**Keywords:** Terminology, Vocabulary, Lexicon, Coding system, Ontology, Interoperability, Semantic computation

## Abstract

Standard clinical terms, codes, and ontologies promote clarity and interoperability. Within radiology, there is a variety of relevant content resources, tools and technologies. These provide the basis for fundamental imaging workflows such as reporting and billing, and also facilitate a range of applications in quality improvement and research. This article reviews the key characteristics of lexicons, coding systems, and ontologies. A number of standards are described, including International Classification of Diseases-10-Clinical Modification (ICD-10-CM), Current Procedural Terminology (CPT), Systematized Nomenclature of Medicine—Clinical Terms (SNOMED CT), Logical Observation Identifiers Names and Codes (LOINC), and RadLex. Tools for accessing this material are reviewed, such as the National Center for Biomedical Ontology BioPortal system. Web services are discussed as a mechanism for semantic application development. Several example systems, workflows, and research applications using semantic technology are also surveyed.

## Introduction

The need for clear clinical communication in radiology has long been recognized, and terminology is a key determinant of such clarity [1, 2]. Examples of standard imaging terminologies developed to promote clarity include the American College of Radiology (ACR) BI-RADS lexicon [3], the inter-society lumbar disc nomenclature [4] and the Fleischner Society thoracic imaging lexicon [5]. While the focus of such work has historically been on improving communication between individuals, these standards also help to address a widespread need for machines to operate on data based on its meaning. This article reviews several standards which facilitate semantic data interchange in radiology, discusses tools and technologies for working with these standards, and describes several example applications in clinical operations and research.

## Review

Several names are given to systems of standardized terms or concepts. Such a system may be referred to as a “terminology,” a “vocabulary,” or a “lexicon” and any of these may be prefaced with the term “controlled.” For the purposes of this review, all of these names will be considered synonymous, and the name “lexicon” will be preferred. A lexicon, then, is simply a list of terms used to refer to concepts in a given domain. These terms are sometimes accompanied by definitions. Having such agreed-upon terms serves to reduce confusion. A classic example in radiology is the finding of consolidation indicating pneumonia on chest radiographs. While this finding may variously be referred to as an “airspace opacity,” a “parenchymal opacification,” or an “infiltrate;” “consolidation” has been identified as the preferred term [5].

Standard terms may also be assigned codes, which are compact labels intended primarily for use by machines. These codes facilitate machine handling and processing of information. Two ubiquitous coding systems in medicine are the International Classification of Diseases (ICD) system published by the World Health Organization, and the Current Procedural Terminology (CPT) system produced by the American Medical Association. An adaptation of ICD version 10 (i.e., ICD-10 Clinical Modification [6], or ICD-10-CM), together with CPT, underpins much of the medical billing in the USA.

Given terms and codes, it is often desirable to relate terms to one another through semantic relationships. For example, anatomical concepts are readily classified using relationships such as a type hierarchy or a part hierarchy. The concept “humerus” for instance could be recorded as a type of a long bone, and a part of the upper extremity. Or the “mitral valve” could be recorded as a type of a cardiac valve, and a part of the heart. Together, such concepts and relationships form a network which may be termed an “ontology.” Ontologies are a foundational technology for building semantic applications (see for example [7]) with a variety of clinical uses [8–10].

### Content Resources

There are many biomedical lexicons, coding systems, and ontologies which provide clinical content. Here, we describe ICD and CPT in further detail and survey other selected resources which are relevant to radiology (see also Table 1).

**Table 1:** Selected imaging-related semantic content resources

Content resource	Description
ICD-10-CM	An adaptation of the World Health Organization’s International Classification of Diseases (ICD) version 10, ICD-10-CM is used in the USA as a source of diagnostic codes. Within radiology, these codes are associated with the indication for an imaging exam.
CPT	Maintained by the American Medical Association, CPT is used in the USA to bill for medical services and procedures. Most radiology exams correspond to CPT codes in the range 70000 to 79999. Image-guided procedures may also be associated with other CPT codes outside this range.
HCPCS	Maintained by the Centers for Medicare and Medicaid Services (CMS), HCPCS is a superset of billing codes divided into two levels. Level I is the CPT code set. Level II covers services generally outside of Level I.
SNOMED CT	A comprehensive global standard for clinical terminology, maintained by SNOMED International (also known as the International Health Terminology Standards Development Organization, or IHTSDO). Commonly used in electronic health records.
FMA	A widely-referenced, comprehensive ontology for human anatomy, developed at the University of Washington.
LOINC	LOINC is an international standard coding system for clinical observations such as laboratory tests and clinical measurements, maintained by the Regenstrief Institute.
RadLex	An ontology of imaging-related concepts, maintained by the Radiological Society of North America (RSNA). RadLex is used as the basis for the LOINC-RSNA Radiology Playbook.
LOINC-RSNA Radiology Playbook	A structured system for naming and coding imaging exams, jointly maintained by the Regenstrief Institute and RSNA.
ACR RADS Reporting Systems	A collection of structured systems for image interpretation. Examples include BI-RADS for breast imaging, LI-RADS for hepatocellular carcinoma imaging and PI-RADS for prostate MRI.

The ICD-10-CM system provides diagnostic codes. In radiology, these codes are typically used to encode the indication for an imaging exam. ICD-10-CM codes begin with a three-character component called the chapter section, or category. This component consists of a letter followed by two digits. For example, category C18 refers to “malignant neoplasm of colon,” category N21 to “calculus of lower urinary tract,” and category S93 to “dislocation and sprain of joints and ligaments at ankle, foot and toe level.” The category may optionally be followed by a decimal point and one or more additional characters which serve to further specify the concept identified by the category. Code C18.7 indicates “malignant neoplasm of sigmoid colon,” code N21.0 signifies “calculus in bladder,” and code S93.422 indicates “sprain of deltoid ligament of left ankle.”

CPT provides billing codes, and these codes are divided into three groups, category I through III. This discussion will be restricted to category I CPT codes, which describe current medical services provided by physicians and other healthcare providers, and which constitute the primary medical billing codes in the USA. These codes have a five-digit format. Most radiology exams correspond to codes from 70000 to 79999. For example, CPT 71020 is “radiologic examination, chest, 2 views, frontal and lateral,” and CPT 70450 is “computed tomography, head or brain; without contrast material.” These imaging CPT codes may be paired with other codes (i.e., outside the 70000 range) in cases of certain image-guided procedures. For example, image-guided placement of a peripherally inserted central venous catheter might be billed using CPT 36569 for the procedural component, and CPT 77001 for the imaging component. Note that in other cases, a single code outside the 70000 range covers both the procedural and imaging components of an image-guided intervention, as in the case of image-guided biliary drain placement which might be billed using the single CPT code 47533.

CPT is also part of a larger system called Healthcare Common Procedure Coding System (HCPCS, often pronounced “hick-picks”), maintained by the Centers for Medicare and Medicaid Services (CMS). HCPCS itself is divided into two levels. HCPCS level I consists of the CPT code set. HCPCS level II codes describe medical services which are generally outside of CPT. These level II codes may be distinguished from CPT codes by virtue of their format, consisting of a single letter followed by four digits. In some cases, there may be overlap between CPT and HCPCS level II. For example, consider bilateral screening mammography with computer-aided detection (CAD). This was historically billed using the HCPCS level II code G0202. In 2017, a new CPT code was released for this procedure, CPT 77067. As of this writing, however, CMS has advised that billing for this exam should continue to use G0202 rather than the CPT code for technical reasons. (This guidance is subject to change. Readers should consult their institutional billing office for specific coding advice.)

Three other important resources with broad applicability in medicine are Systematized Nomenclature of Medicine—Clinical Terms (SNOMED CT), the Foundational Model of Anatomy (FMA) and Logical Observation Identifiers Names and Codes (LOINC). SNOMED CT is a comprehensive, multilingual, international standard system of clinical terms, with more than 330,000 concepts. This system represents a combination of works by the College of American Pathologists, and the United Kingdom’s National Health Service. SNOMED CT is now maintained by an organization called SNOMED International, also known as the International Health Terminology Standards Development Organization (IHTSDO). SNOMED CT includes content in areas such as clinical signs and symptoms, infectious agents, medications, and medical devices, among many others. SNOMED CT is widely used in electronic health records.

The FMA is a reference ontology of human anatomy, which models the human body at several scales, ranging from the macromolecular scale to the organism scale. The FMA contains approximately 75,000 anatomic concepts and over 2.1 million relationships. LOINC is an international standard system for clinical observations, widely used for laboratory tests among other areas. LOINC is published by the Regenstrief Institute, and has approximately 85,000 codes.

In radiology, the RSNA RadLex ontology [11–13] aims to provide a comprehensive resource for imaging-related terms, spanning areas such as imaging technologies, imaging findings, anatomy, and pathology. RadLex contains more than 45,000 concepts, and assigns each of these a unique code (i.e., a RadLex identifier, or RID) as well as a preferred name. Synonyms or translations may also be attached to each concept. For example, RID4271 has the preferred name “hepatocellular carcinoma” and the synonyms “HCC” and “hepatoma.” Work has also been done by the Society for Imaging Informatics in Medicine (SIIM) to formalize a lexicon of radiology workflow terms known as SIIM’s Workflow Initiative for Medicine (SWIM) [14]. Also note that recent collaboration between RSNA and the Regenstrief Institute has led to the LOINC-RSNA Radiology Playbook [15], a system for naming and coding imaging exams, constructed using RadLex terms. This system builds on previous work embodied by the RadLex Playbook, and what was previously known as LOINC Radiology.

In addition to the BI-RADS system mentioned above, the ACR has also developed a number of other standardized reporting systems in specific clinical areas, each with its own lexicon. These include the Lung-RADS system for lung cancer screening CT, the LI-RADS system for hepatocellular carcinoma imaging, the PI-RADS system for prostate MRI and the C-RADS system for CT colonography [16]. Codes for many of the terms and categories defined by these reporting systems may be found in RadLex.

### Interoperability and Semantic Computation

To the extent that lexicons, coding systems, and ontologies are represented in machine-processable forms, this content becomes computationally accessible. That is, computer systems can parse and use the terms, codes, concepts, and relationships contained therein. The purpose of computational clinical content resources is to enable systematic processing of healthcare information, in ways which leverage the meaning of that information. Semantic interoperability has been defined as the ability of computers “to share, understand, interpret and use data without ambiguity” [17], and alternatively as the ability of machines “to collect, process, analyze or exchange comparable data elements on the basis of the meaning of the data” [15]. (A complementary concept is that of syntactic interoperability, or the ability of machines to transmit data without regard to its meaning. This relates to issues of formatting and communications protocols, and will not be the focus of this discussion.) For example, a facility wishing to collect a local registry of colon cancer patients could begin by searching for medical records with the ICD-10-CM diagnostic category C18 for colon malignancy. This simple aggregation of patients with this shared diagnostic code represents a form of semantic interoperability between these records.

Semantic resources may also be used to compute new information, based on a set of given facts. This may be referred to as “machine reasoning,” “knowledge inferencing,” or “semantic computation.” For example, consider an anatomic ontology which states that Couinaud segment 2 is part of the left hepatic lobe, and separately that the left hepatic lobe is part of the liver. Although not stated explicitly, it may be inferred that the liver has segment 2 as one of its parts. Semantic computation is a component technology in natural language processing among other application areas.

### Tools and Technologies

There are a variety of tools and technologies for accessing and using imaging-related content resources. The Unified Medical Language System (UMLS) is a collection of over 200 biomedical content resources maintained by the National Library of Medicine (NLM) [18]. UMLS distributes SNOMED CT at no cost under the NLM license for use within the USA. Note that users in non-member countries are charged with a license fee by SNOMED International. UMLS also provides access to ICD and CPT, among many other resources.

Another important repository of biomedical content resources is the BioPortal system hosted by the National Center for Biomedical Ontology (NCBO) [19]. BioPortal provides access to over 600 biomedical ontologies, including SNOMED CT, ICD, CPT, FMA, and RadLex.

Both UMLS and BioPortal provide tools for working with semantic content. One useful tool is the BioPortal Annotator. This tool accepts an arbitrary block of free text as input. The user may also specify one or more ontologies of interest. The Annotator tool then finds all matches in the given text to concepts in the selected ontologies, or the entire corpus of ontologies if none are specified, with options to account for factors such as synonyms and partial matching.

UMLS and BioPortal also both provide web services interfaces to their content and tools. Web services are a common technology for building software applications utilizing functionality provided by remote servers. This technology has entered the clinical realm in recent years, as exemplified by the HL7 Fast Healthcare Interoperability Resources (FHIR) specification [20] and the DICOMweb portion of the DICOM standard [21]. Web services allow developers to rapidly and programmatically combine existing data and processing resources to create new applications. In the realm of ontologies, the UMLS and BioPortal web service interfaces can be used to access semantic content for further computation.

While the BioPortal web service interface allows for term searching and matching operations, more sophisticated queries may be accomplished with a dedicated ontology query language. SPARQL [22] has been identified by the World Wide Web Consortium (W3C) as a standard ontology query technology, and web-based implementations of SPARQL query engines have been described [23].

**Fig. 1:**
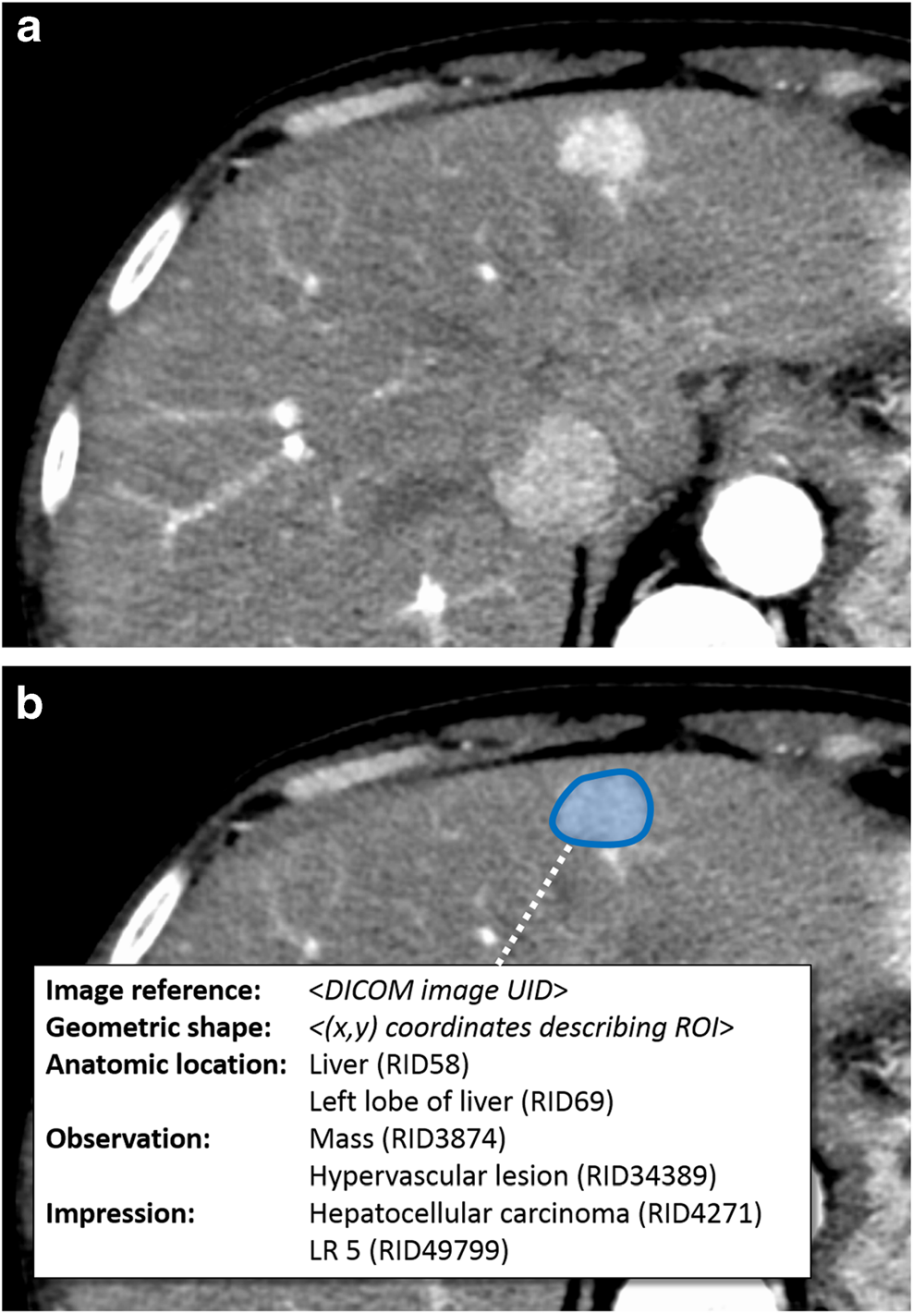
Semantic image annotation with AIM. A portion of an axial CT image through the liver after intravenous contrast in arterial phase (**a**) demonstrates a hypervascular mass in the left hepatic lobe. While a report would describe this lesion, semantic image annotation using the AIM standard (**b**) enables the findings and diagnosis (white box, inset) to be associated with a specific region of interest (ROI) within the image (blue). AIM uses the extensible markup language (XML) to group a reference to the relevant image (through a DICOM unique identifier, or UID), a geometric description of the ROI, and semantic information such as anatomic location, imaging observations and diagnostic impressions. Such annotations allow the geometric and imaging characteristics of the lesion to be incorporated into other computation. Here for example, the pixel data within the ROI could be quantified to study the enhancement texture characteristics of hepatocellular carcinoma

Finally, radiologic information is sometimes divided into two components, the “words” and the “pixels,” or the reports and the images. The Annotation and Image Markup (AIM) standard [24, 25] bridges this divide by allowing semantic concepts to be associated with regions of an image. Using AIM, the pixels of an imaging finding may be tagged with conceptual codes from an ontology (Fig. 1). Just as codes facilitate semantic interoperability and semantic computation based on text reports, so AIM associations permit the imaging data itself to be incorporated into semantic operations and machine learning systems (see for example [26] for related work on texture analysis of brain tumors). The ePAD project provides one freely available implementation of AIM functionality [27].

## Discussion

Coded concepts, as described by these content resources, have a fundamental role to play in the capture of information from radiology reports and images. Such capture from reports may occur at the time of report generation, through coded structured reporting templates and common data elements [28, 29]. Or, semantic capture from reports may be done as a post-processing step in the form of natural language processing of free text reports [30]. Semantic capture from images may be achieved with annotation technologies such as AIM. Some combination may also be used, as these approaches are not mutually exclusive. Regardless of the method, the objective of such capture is to encode the information contained in radiology reports and images for use in processing and computation.

**Fig. 2:**
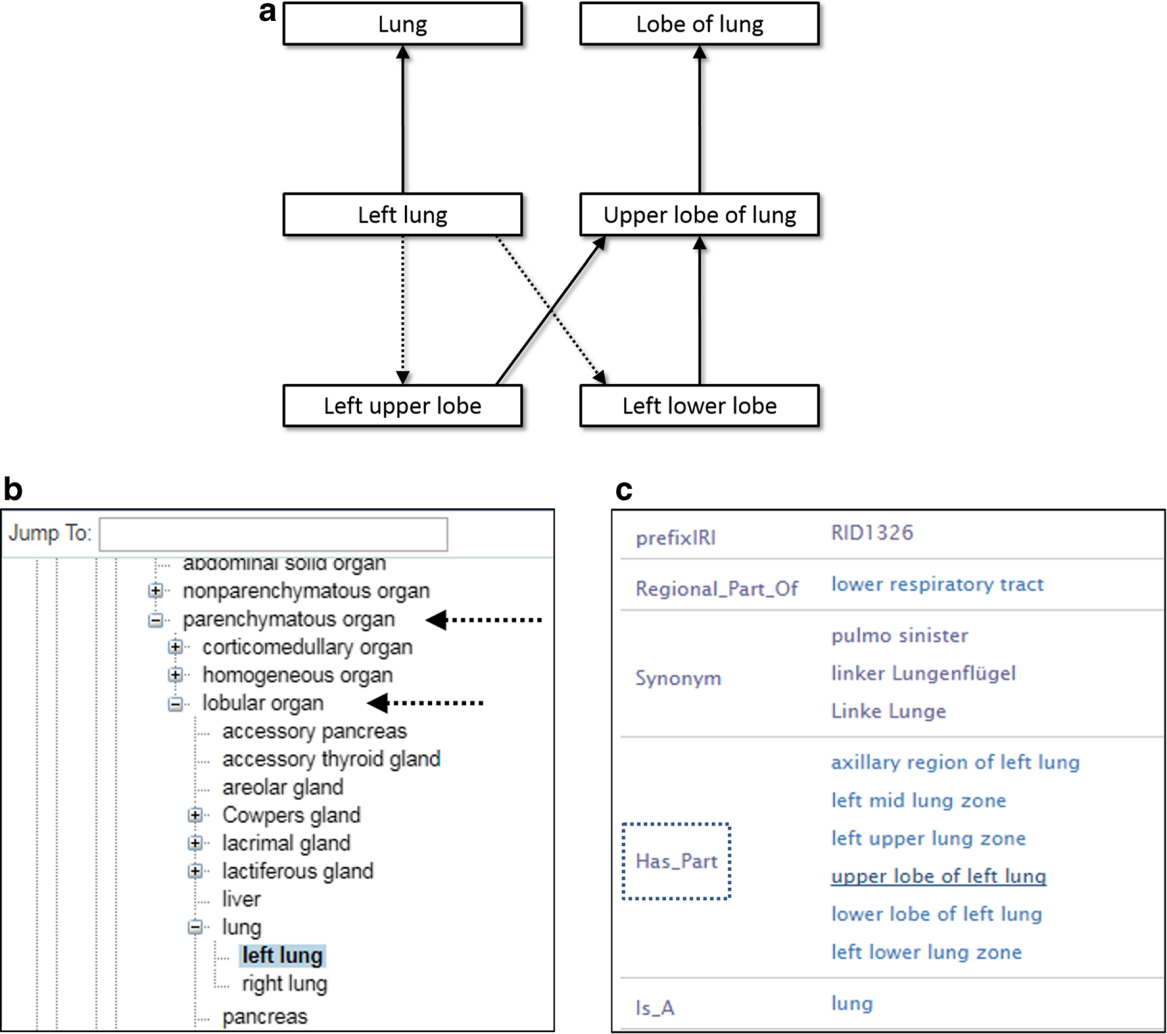
The type hierarchy and part relationship in RadLex. RadLex is organized fundamentally by the Is-A hierarchy, or type hierarchy. By this principle, RadLex terms are encoded as a type of some higher level concept. RadLex concepts may also have other kinds of relationships, such as Has-Part, Has-Member, and Has-Innervation-Source. Consider the left lung and its lobes (**a**). Here, Is-A relationships are shown as solid arrows, and Has-Part relationships are shown as dotted arrows. The left lung is a type of lung, and therefore possesses the Is-A relationship to lung. The left lung also Has-Part “left upper lobe” and Has-Part “left lower lobe”. The lobes themselves, however, possess the Is-A relationship to “upper lobe of lung” which itself Is-A “lobe of lung.” Graphical browsers such as the BioPortal interface (portions shown here in panels **b** and **c**) display RadLex terms in an expandable tree (**b**) of concepts based on this Is-A hierarchy, together with concept metadata (**c**). Here, “left lung” (highlighted in blue) is shown as a subclass of “lung” (**b**) (itself a subclass of “lobular organ” which is in turn a subclass of “parenchymatous organ,” dotted arrows). The lobes of the left lung are shown in **c** via the Has-Part relationship (dotted box). The lobes of the left lung are not accessible by expanding the tree (**b**), since the lobes are not types of the left lung

In order to find relevant codes in content resources, it is useful to develop familiarity with the organization of those resources. Consider RadLex as an example. RadLex is an ontology organized fundamentally along a type hierarchy, also called the Is-A hierarchy. This defines parent (i.e., class) and child (i.e., subclass) relationships, where a given subclass is a kind or a type of its parent. For example, the RadLex concept “left lung” (RID1326) is a subclass of RadLex concept “lung” (RID1301), because “left lung” is a type of “lung.” Note, however, that “left lung” does not have subclasses “left upper lobe” or “left lower lobe,” because neither of these is itself a type of “left lung.” Rather, these lobes are parts of the left lung and are encoded using the Has-Part relationship (e.g., “left lung” Has-Part “left upper lobe”). Note that “left upper lobe” (RID1327) is itself a subclass of “upper lobe of lung” (RID34695) which is in turn a subclass of “lobe of lung” (RID34694), because “left upper lobe” is a type of “upper lobe of lung” which is a type of “lobe of lung” (see Fig. 2).

The organization of RadLex by the Is-A hierarchy is important because graphical interfaces to RadLex content [13, 19] display RadLex concepts based on this hierarchy. Users may find that this leads to unexpected results. For example, consider RadLex concept “set of BI-RADS terms” (RID34298). This has a subclass “set of BI-RADS mammo terms” (RID34277). Users may expect to find children of “set of BI-RADS mammo terms” representing BI-RADS imaging descriptors. However, “set of BI-RADS mammo terms” currently has no subclasses, since there are no further specified types of term sets. Rather, “set of BI-RADS mammo terms” is associated with other RadLex concepts through the Has-Member relationship, so that terms like “architectural distortion” (RID34261), “dystrophic calcification” (RID34246), and “spiculated margin” (RID5713) are all members (not subclasses) of “set of BI-RADS mammo terms.”

**Fig. 3:**
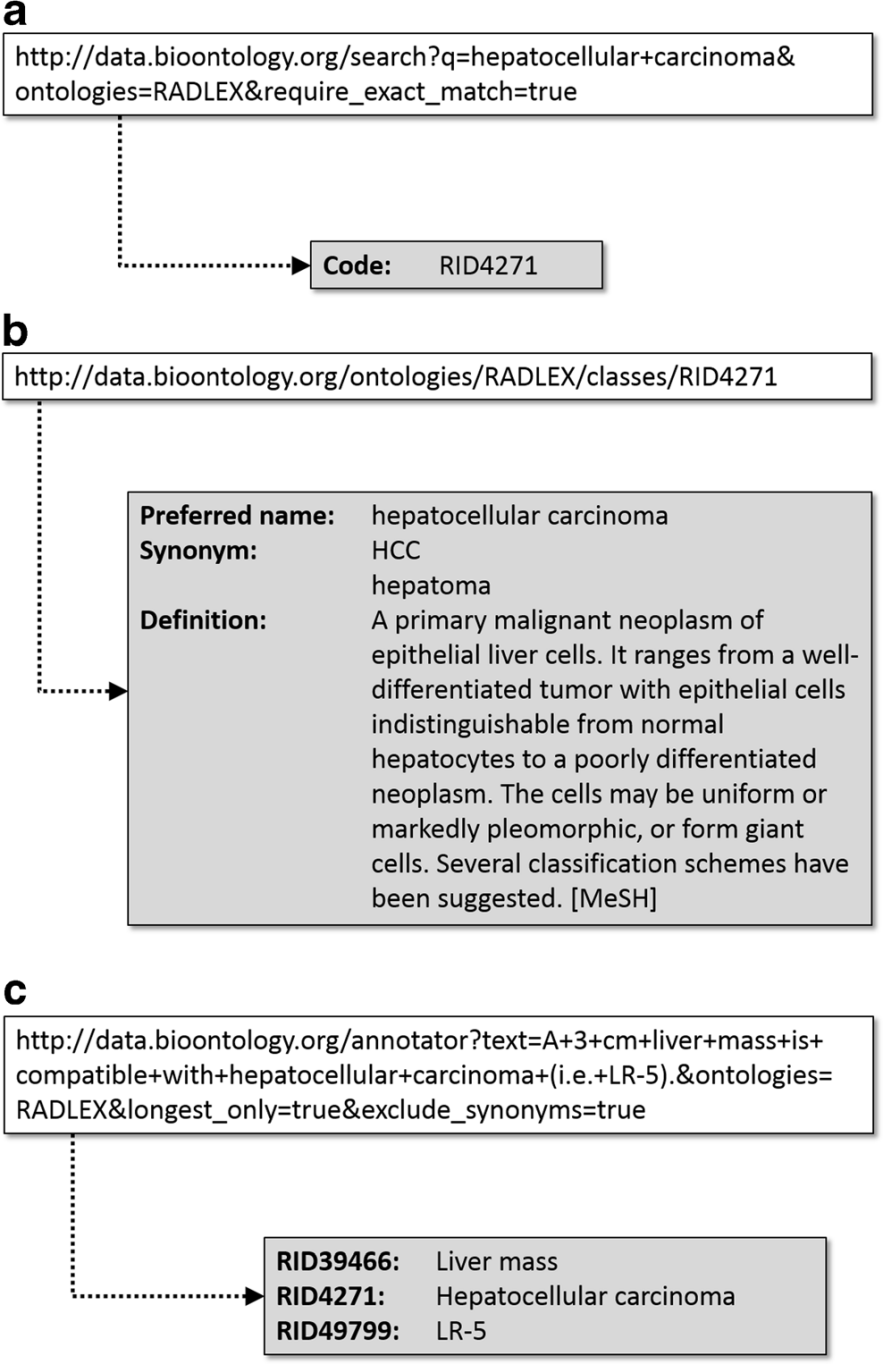
Example web service calls using BioPortal and RadLex. The BioPortal web service application programming interface (API) allows programmatic access to ontology content and semantic tools. Operations are invoked by a client using http requests (white box), and the server responds with data encoded in the JSON format (edited and shown in the shaded box). These results may then be used by the client to perform further computation. In **a**, a search for the term “hepatocellular carcinoma” within RadLex is requested, leading to the result RID4271, the code of the corresponding RadLex concept. In **b**, retrieval of that RadLex concept is requested, leading to the associated metadata shown. In **c**, the BioPortal Annotator tool is invoked on the input text, “A 3-cm liver mass is compatible with hepatocellular carcinoma (i.e., LR-5).” The result of this request is the set of RadLex concepts identified in the text, consisting of RID394666 (liver mass), RID4271 (hepatocellular carcinoma), and RID49799 (the LI-RADS assessment category LR-5). Note that the http requests shown here omit the required user identifier (and which may be obtained with free BioPortal registration)

Using the tools and technologies surveyed above, semantic data from reports and images may be combined with other clinical data and ontologic information to build semantic applications. Web services offer a convenient way to approach such application development, as mentioned above. The term “web services” refers to a group of technologies which can utilize the ubiquitous Hypertext Transfer Protocol (HTTP) communication protocol to allow programmatic interaction between programs and servers. One popular form of web service technology is known as Representational State Transfer (REST or RESTful). A simple example RESTful transaction consists of an HTTP GET operation to request data, with the responding server returning the result in Javascript Object Notation (JSON) form (see Fig. 3). JSON is a simple text-based data format, which can be easily parsed by the receiving program. Furthermore, while Javascript is a convenient language to use for this type of transaction, any programming language or scripting language capable of handling HTTP operations could be used. Web services allow developers to rapidly leverage server-based capabilities such as ontology search, text annotation and image handling. Note for example that RESTful technology is part of the SIIM Hackathon platform [31]. See Fig. 3 for sample web service calls using BioPortal and RadLex.

Semantic technologies have been used to construct a variety of different applications and workflows in radiology. The use of ontologies to build imaging differential diagnosis systems has been described [32, 33]. Semantic tagging of imaging and pathology reports has been used to automate radiologic-pathologic follow-up, by matching biopsy results with preceding imaging exams [34].

Abajian et al. described a system for lesion tracking, based on AIM annotations [35]. These annotations permit quantitative lesion measurements (such as diameter, circumference, and area) to be systematically tracked across serial imaging exams. Zimmerman et al. developed a system for automatically populating a structured reporting template with quantitative AIM measurement data [36]. In other work, AIM annotations were used to build an imaging atlas, which draws upon ontologic relationships to facilitate image navigation [37].

The semantics of radiology procedure codes has also been used to drive quality improvement efforts. The ACR Dose Index Registry (DIR) is a national registry which enables participating institutions to compare computed tomography (CT) dose indices with those from other facilities [38]. DIR uses procedure codes constructed with RadLex terms to receive CT dose data. By virtue of their component terms, these procedure codes may be aggregated to permit apples-to-apples comparison of dose information. Procedure code semantics have also been used to identify unnecessary repeat imaging within a health information exchange [39].

**Fig. 4:**
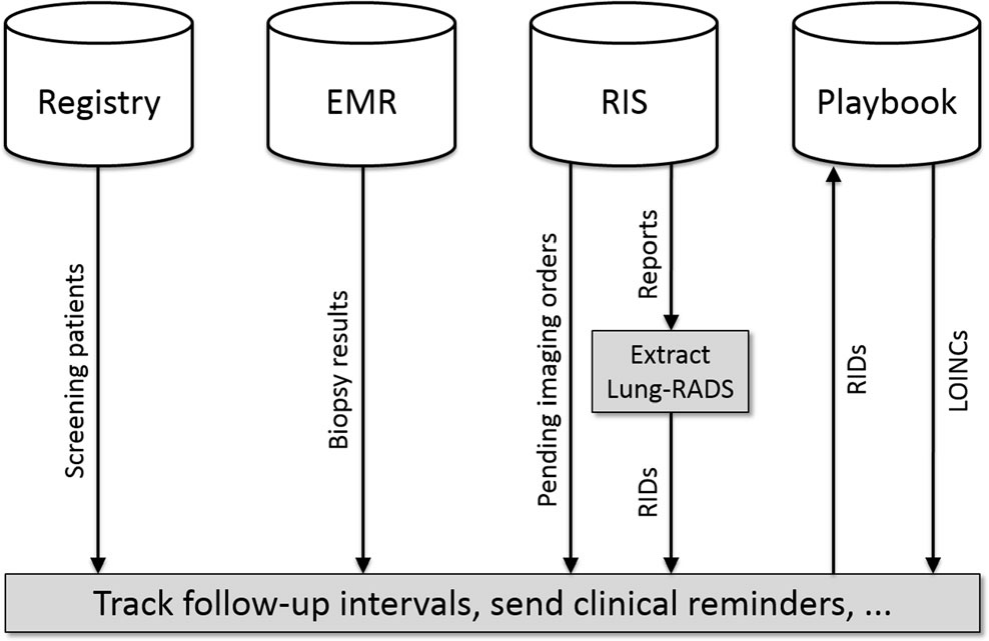
Information flow and the role of codes in a lung cancer screening system. Lung cancer screening programs depend on a variety of factors, such as clinical risk factors, dedicated imaging protocols, imaging reports, and pathology data. Here, a sample system is illustrated, for automated tracking of screening patients. A registry provides a listing of patients in a screening program. An electronic medical record (EMR) provides clinical data, such as biopsy results. A radiology information system (RIS) provides information about a patient’s imaging history (i.e., previous imaging reports) as well as pending imaging orders. Extraction of Lung-RADS assessment categories from imaging reports leads to coded concepts in the form of RadLex identifiers (RIDs). A procedure coding system such as the LOINC-RSNA Radiology Playbook provides a source of structured exam codes (LOINCs) relevant to lung imaging (which may be computed based on given exam characteristics specified using RIDs). These inputs and codes would then enable automated functions such as lung nodule follow-up tracking (i.e., is a patient undergoing follow-up imaging at appropriate intervals?), handling clinical reminders (i.e., notifying responsible physicians if a target follow-up interval has lapsed without an imaging order or exam), biopsy tracking (i.e., if a biopsy has been recommended, has it been done?) and report quality monitoring (i.e., do screening CT reports routinely give Lung-RADS assessments?)

As another example, consider the problem of lung cancer screening. This requires continuous monitoring of screening patients, relevant imaging, and other clinical data. Inputs to this process include a registry of screening patients, clinical information such as biopsy results, previous imaging reports, pending imaging orders, and target follow-up intervals (see Fig. 4). When these inputs are processed in terms of codes such as Lung-RADS assessment categories, LOINC-RSNA Radiology Playbook codes, and RadLex concepts, this facilitates robust automation of functions such as tracking nodule follow-up, monitoring report quality, and sending clinical reminders for imaging orders when necessary.

In the future, the use of semantics in clinical data processing and exchange promises to expand further, facilitating continued advances in efficiency, precision, and quality in imaging.

## Summary

There are many resources, tools, and technologies available for developing semantic applications in radiology. Codes facilitate machine processing of concepts. Ontologies provide information about the relationships between concepts. Web services constitute an important technology for semantic application development, and a variety of such applications have been described in the literature.
